# Analyzing the Simonshaven Case With and Without Probabilities

**DOI:** 10.1111/tops.12436

**Published:** 2019-07-25

**Authors:** Bart Verheij

**Affiliations:** ^1^ Department of Artificial Intelligence Bernoulli Institute, University of Groningen

**Keywords:** Evidential reasoning, Probabilistic models, Argumentation models, Narrative models, Case analysis, Rational proof, Criminal law

## Abstract

This paper is one in a series of rational analyses of the Dutch Simonshaven case, each using a different theoretical perspective. The theoretical perspectives discussed in the literature typically use arguments, scenarios, and probabilities, in various combinations. The theoretical perspective on evidential reasoning used in this paper has been designed to connect arguments, scenarios, and probabilities in a single formal modeling approach, in an attempt to investigate bridges between qualitative and quantitative analytic styles. The theoretical perspective uses the recently proposed logical formalism of case models, where cases represent possible combinations of evidence and events, ordered by an ordering relation. In the context of evidential reasoning, the ordering relation can be represented by a probability function. As an ordering relation is qualitative in nature, the theoretical perspective is in a formally precise sense simultaneously with and without probabilities.

## Abstract 2

Verheij analyzes the case with his case model formalism, which connects arguments, scenarios and probabilities to bridge quantitative and qualitative analytic styles. Thus, probabilistic reasoning errors may be prevented without having to express probabilities on all elements of a model. Verheij claims that his approach is natural in that it models the gradual development and evaluation of scenarios in light of evidence. However, its mathematical nature may make it hard for legal professionals to apply it.

## 1 Introduction

**Figure 1:**
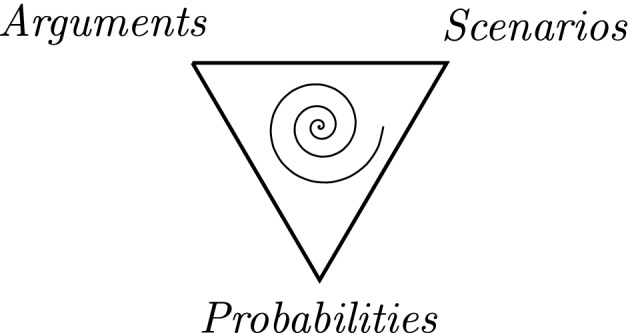
Arguments, scenarios, and probabilities.

The analysis of the Simonshaven case developed in this paper uses a theoretical perspective on evidential reasoning “with and without probabilities.” Instead of choosing between one of the three primary analytic tools discussed in the literature, namely arguments, scenarios, and probabilities (Anderson et al., 2005; Dawid et al., 2011; Di Bello & Verheij, 2018; Kaptein et al., 2009), the theoretical perspective used in this paper has been designed to connect arguments, scenarios, and probabilities in one approach, in an attempt to bridge qualitative and quantitative analytic styles (Fig. 1) (Verheij, 2014, 2017).

In this way, an attempt is made to use a single framework to study themes that are investigated separately in each of the approaches. For instance, in argumentative approaches—for criminal law going back to Wigmore (1913)—argument structure, defeat, and evaluation are studied (Bex et al., 2003; van Eemeren et al., 2014; Gordon & Walton, 2009; Prakken & Sartor, 2009; Walton, 2002). In scenario approaches—influential in legal psychology (Bennett & Feldman, 1981; Crombag et al., 1992; Keppens & Schafer, 2006; van Koppen, 2012; Pennington & Hastie, 1993; Wagenaar et al., 1993)—sets of events are considered as coherent wholes and analyzed in connection with the evidence. In probabilistic analyses—going back to early forensic science (Taroni et al., 1998)—it is made explicit how hypothethical events are probabilistically connected to the evidence. Combined approaches are being studied, for instance, inference to the best scenario (Allen & Pardo, 2007), scenarios and arguments (Bex, 2011; Bex et al., 2010), evidential Bayesian networks (Fenton et al., 2013; Hepler et al., 2007; Keppens, 2012), and scenarios and probabilities (Di Bello, 2013; Urbaniak, 2018). See Di Bello and Verheij (2018) for further detail about the three approaches and their connections.

Formally, the theoretical perspective in this paper uses case models (Verheij, 2017), formalizing the modeling style of evidential reasoning developed in (Verheij, 2014). A case model consists of cases that are expressed by logical sentences of what can be the case, ordered by a preference ordering.

For analyzing evidential reasoning, each case in a case model can be thought of as representing a possible combination of evidence and a hypothetical cluster of events, such as a scenario. The preference ordering represents which cases are equivalent or preferred over other cases. Here, the ordering can be interpreted in different ways, depending on the use of the formalism. For instance, in an argumentation perspective, the ordering can be used to model the validity, defeat, and (relative) strength of arguments. In a scenario perspective, the ordering can be used to model quality properties of scenarios, like consistency, plausibility, and completeness. In a probabilistic perspective, the ordering can be used to model relative probability, positive probability, and probability above a threshold.

The ordering on cases in a case model is formally a total preorder; hence, it provides the kind of ordering that can be represented by a numeric representation. Therefore, in a formally precise sense, the ordering on cases is simultaneously with and without numbers: It is with numbers since the ordering can be derived from a numeric representation; it is without numbers since an ordering is a qualitative relation. In the context of evidential reasoning, a numeric representation of the ordering can be taken to be a probability function.

The approach is inspired by findings in a Netherlands‐based research project on analyzing evidential reasoning using Bayesian networks. In the project, methods have been developed for the design and understanding of Bayesian networks using scenarios (Vlek, 2016; Vlek et al., 2016) and using arguments (Timmer, 2017; Timmer et al., 2017). The methods developed build on earlier work on the use of Bayesian network modeling for evidential reasoning (Fenton et al., 2013; Hepler et al., 2007).

While developing the methods in the project, well‐known issues with Bayesian network modeling were encountered. The first issue concerns the incompleteness of numeric information. The issue arises since a Bayesian network model is a representation of a complete joint probability distribution over the variables used in the model, whereas it is not straightforward to establish or estimate all numbers that are needed. The second issue concerns transparent interpretation. The issue arises since, although the formal meaning of a Bayesian network model is well‐defined, there is the risk of misinterpreting the graphical structure of a Bayesian network, for instance in causal terms (Dawid, 2010).

The theoretical perspective of case models used in this paper has been designed to address these issues. For addressing the issue of incompleteness of numeric information, the case model approach does not require the representation of a complete joint probability distribution over the variables. For addressing the issue of transparent interpretation, the case model approach uses notions of formal validity to evaluate arguments and scenarios.

Section 22 provides background materials on the Simonshaven case and the case model formalism. In Section 32.2.1, the analysis of the case is developed. Section 43.2 provides the full formalized case model. The paper ends with discussion and conclusion sections.

## 2 Background

Section 2.12.1 provides an overview of the case, used as an illustration of the case model formalism in connection with arguments, scenarios, and probabilities (Section 2.22.2).

### 2.1 Overview of the case

At first instance (Court of Rotterdam, November 30, 2012), during the discussion at court, two possible hypotheses about what might have happened were considered: the guilty‐as‐charged hypothesis—presented by the prosecution—that Ed Lourens killed Jenny Lourens, and the robbery hypothesis—presented by the defence and based on Ed’s statement—that Jenny was killed by a robber jumping from the bushes. There also is a third, not explicitly discussed, but analytically relevant complimentary possibility that neither the guilty‐as‐charged scenario nor the robbery scenario is true, and that something different has happened. At the first instance, it was decided that the guilty‐as‐charged scenario was proven lawfully and convincingly. In other words, the other two hypothetical possibilities were decided to be (sufficiently) excluded by the evidence.

At the second instance (Court of Appeal The Hague, February 18, 2015), the robbery hypothesis was split into two variations of what might have happened. One version of the robbery hypothesis is the Perry scenario, in which Perry Sultan is considered to be the killing robber, perhaps helped by Tom Seelen. The other version is the scenario in which there is another unknown third person who is the killing robber. The court again decided that the guilty‐as‐charged scenario was proven lawfully and convincingly. The other three hypothetical possibilities were excluded by the evidence.

**Figure 2:**
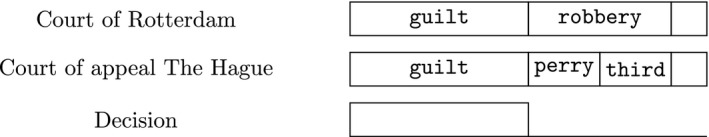
Overview of the case.

In Fig. 2, the hypothetical possibilities as considered by the two courts are graphically shown as boxes. At the top, the three options as considered by the Court of Rotterdam are shown.

1. the guilty‐as‐charged hypothesis that Ed Lourens is guilty (marked guilt);
2. the robbery hypothesis that Jenny was killed by a robber (marked robbery); and
3. the complementary hypothesis that something different has happened.

At the Court of Appeal The Hague, the second option is considered in two variations:

**Table:** 

2.	a.	The hypothesis that Perry was the killing robber (marked perry);
	b.	the hypothesis that a third person, not Ed, not Perry, was the killing robber (marked third).

In both decisions, the guilty‐as‐charged scenario was considered to be proven by the evidence, as graphically indicated at the bottom of the diagram: the guilty‐as‐charged scenario is selected and the other hypotheses are excluded, as indicated by the line.

### 2.2 Case models and their connection to arguments, scenarios, and probabilities

Here, an informal introduction of case models is provided, as needed for the case analysis developed in this paper. Details of the case model formalism are given in Verheij (2017). Formally case models consist of consistent mutually exclusive sentences, ordered by a preference ordering. The four hypotheses discussed in Section 2.1 can be formalized as cases as follows:

**Table:** 

1.		guilt
2.	a.	­guilt ∧ robbery ∧ perry
	b.	­guilt ∧ robbery ∧ ­perry ∧ third
3.		­guilt ∧ ­robbery

Here, ­ represents logical negation and ∧ logical conjunction, so option 2a means “Ed Lourens is not guilty as charged and Jenny was killed in a robbery and Perry is the robber.” Option 3 means “Ed Lourens is not guilty as charged and Jenny was not killed in a robbery,” representing the situation that the case of Jenny’s murder is not solved as in the guilty‐as‐charged and robbery hypotheses. Note that guilt represents Ed’s guilt as charged by the prosecution, and not his guilt of killing Jenny in other ways. Another, as yet unknown, way in which Ed would be guilty of killing Jenny would fall under the third hypothesis.

These cases do not yet represent the evidence, which for now, we will logically summarize as all‐evidence, representing the totality of the evidence. In the full analysis below (Section 3), details of the actual evidence are modeled in a more specific case model.

Following the court decisions, only option 1 is compatible with the totality of the evidence, and the other options are not compatible with all the evidence. Formally we can model this by adapting the formulas above. For case 1, we add ∧ all‐evidence, for the others ∧ ­all‐evidence:

**Table:** 

1.		guilt ∧ all‐evidence
2.	a.	­guilt ∧ robbery ∧ perry ∧ ­all‐evidence
	b.	­guilt ∧ robbery ∧ ­perry ∧ third ∧ ­all‐evidence
3.		­guilt ∧ ­robbery ∧ ­all‐evidence

These four cases can be thought of as representing a brief summary of the hypotheses and the evidence considered by the Court of Appeal in The Hague. According to this summary, options 1, 2a, 2b, and 3 are the four possibilities considered in the case proceedings. Of these, only the first is compatible with all the evidence, so given all the evidence, the guilty‐as‐charged hypothesis is legally proven and the other options are legally excluded. Graphically, this is shown in Fig. 2 by the bottom line showing the decision where of the four boxes only the one representing the guilty‐as‐charged hypothesis has remained, and the others have been excluded (suggested by the line).

Case models also contain a preference ordering, formally a total preorder, that is, reflexive and transitive. For instance, let us assume that the cases above can be ordered as follows:

1 > 2a ~ 2b > 3

Diagrams can be used as graphical illustration of case models. The formal case model discussed here corresponds to the four boxes modeling the hypotheses considered by the Court of Appeal in Fig. 2. The ordering of the four cases is reflected in the relative sizes of the boxes: Option 1, the guilty‐as‐charged hypothesis, is ordered higher than the two robbery hypotheses 2a and 2b, which are ordered equally, and in turn are ordered higher than option 3 that something different has happened. The absolute sizes of the boxes have no counterpart in the formal case model.

In evidential reasoning with arguments, scenarios, and probabilities, cases and their ordering can be interpreted in different ways, as follows. Formal details of the terminology used are given in Verheij (2017).

#### 2.2.1 Arguments

Arguments from premises to conclusions can be formally evaluated given a case model using three kinds of formal argument validity: coherence, presumptive validity, and conclusiveness.

An argument from premises to conclusions is *coherent* when there is a case in which both the premises and the conclusions hold. In a sense, coherence models which conclusions are possible given the premises. For instance, in the case model above the argument from the evidence to the guilty‐as‐charged hypothesis is coherent, since in Case 1 both all‐evidence and guilt obtain. The argument from the evidence to the Perry hypothesis is not coherent since there is no case in which both all‐evidence and perry obtain.

A coherent argument is *presumptively valid* when the argument’s conclusions obtain in a maximally ordered case in which the premises obtain. One could say that presumptive validity models default conclusions given the premises. Such default conclusions can be defeated given further information. For instance, the argument from empty premises (which obtain in all cases and are logically formalized as a tautology) to the guilty‐as‐charged hypothesis is presumptively valid, since Case 1 is maximal in the ordering. The argument from empty premises to the Perry hypothesis is not presumptively valid, since Case 2a is not maximal in the ordering.

A coherent argument is *conclusive* if the argument’s conclusions obtain in all cases in which the premises obtain. Conclusive arguments model a kind of certain reasoning. For instance, the argument from the evidence to the guilty‐as‐charged hypothesis is conclusive since the only case in which all‐evidence obtains is Case 1 and in that case also guilt obtains. Case models can model argument defeat. For instance, the presumptively valid argument from the empty premises to the guilty‐as‐charged hypothesis has ­all‐evidence as defeating circumstances, since the argument is no longer presumptively valid after adding ­all‐evidence to the argument’s premises. Since the argument from ­all‐evidence to ­guilt, the negation of the defeated argument’s conclusion, is presumptively valid, the defeating circumstances are rebutting, in the terminology proposed by Pollock (1987).

#### 2.2.2 Scenarios

Scenarios represented by structured logical sentences can be evaluated using case models. Three kinds of evaluation are distinguished, following the kinds of logical validity discussed above.

A scenario is *coherent* when there is a case in the case model that logically implies the scenario. So the robbery scenario in which Perry is the robber killing Jenny, represented by the logical sentence robbery ∧ perry, is coherent since Case 2a logically implies the sentence.

A coherent scenario is *plausible* when it is implied by a presumptively valid case (i.e., it is a case that is maximal in the ordering). So the guilty‐as‐charged scenario, here represented simply as guilt, is plausible since it is implied by Case 1 that is maximal in the ordering.

A coherent scenario is *beyond a reasonable doubt* when it is implied by all cases. Here, there is no scenario beyond a reasonable doubt.

The three kinds of evaluating scenarios can be applied *relative to given evidence*. For instance, a scenario is coherent given the evidence, when there is a case that implies the scenario and that also implies the evidence. Here, only the guilt is coherent given all the evidence all‐evidence. A coherent scenario is plausible given the evidence when there is a case that implies the scenario that is maximal in the ordering among the cases that imply the evidence. Here, the guilt scenario is plausible given all the evidence all‐evidence. A coherent scenario is beyond a reasonable doubt given the evidence when it is implied by all cases that imply the evidence. Indeed, the guilt scenario is beyond a reasonable doubt given all‐evidence.

#### 2.2.3 Probabilities

The kinds of validity can be given a probabilistic interpretation. Formally this is possible since the ordering of a case model is a total preorder, which are the kinds of ordering that can be represented numerically, even probabilistically. For instance, using the sizes of the boxes for choosing the numbers, we can assign Case 1, Case 2a, Case 2b, and Case 3, probabilities 50%, 20%, 20%, and 10%, respectively. Clearly, these numbers are just one of many possibilities that could numerically realize the ordering Case 1 > Case 2a ∼ Case 2b > Case 3 with positive probabilities. If we would be able to provide good probabilistic estimates of how probable each case is (which often will not be the case), we could use those. The current choices represent that the guilty‐as‐charged hypothesis is modeled as most probable, followed by the two robbery hypotheses, considered as equally probable, in turn followed by the even less, but still positively probable “something else has happened” option.

Of the three kinds of validity, coherence corresponds to *positive probability*. Here all three cases have positive probability, so they are coherent. More generally, the strength of an argument can be measured by the conditional probability of the conjunction of the argument’s premises and conclusions conditioned on that of the premises. So the argument from empty premises to guilt has strength 50/100 = 50% = 0.5 and the argument from all‐evidence to guilt has strength 50/50 = 100% = 1. It can be proven that presumptive validity can be characterized by *strength above a threshold*. Conclusiveness can be characterized as *strength equal to 1*, as, for example, the argument from all‐evidence to guilt.

## 3 Analysis of the Simonshaven case using case models

In this section, the analysis of the Simonshaven case is developed as a series of ever more specific case models, illustrated with diagrams. Section 3.1 handles the initial investigation, Section 3.2 the alternative robbery scenario (in two variations), Section 3.3, the statement of the suspect (Section 3.3), and Section 3.4 the suspect’s wounds and his physical capabilities. The full formalized case model of the Simonshaven case is given in Section 4.

### 3.1 Initial findings

**Figure 3:**
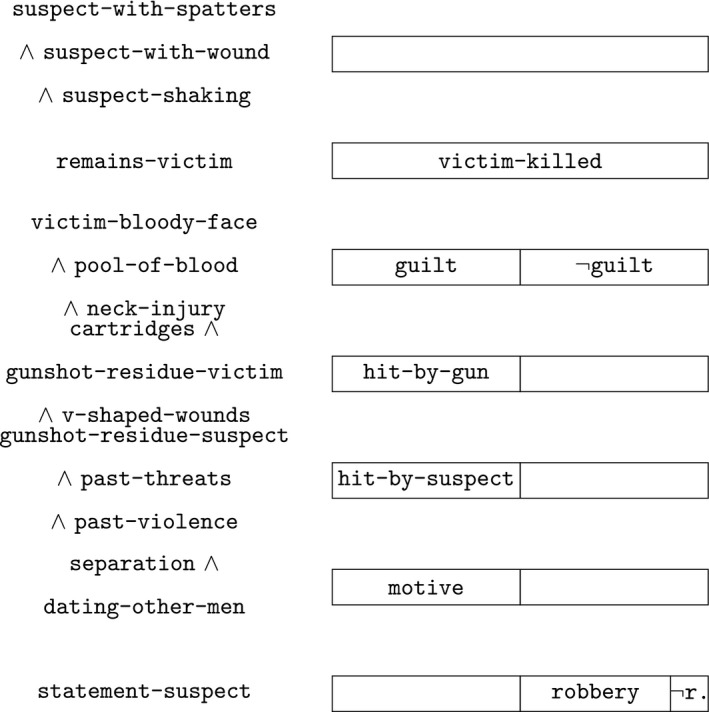
The initial findings: A guilt suspect or a robbery.

When the police arrive on the scene, they find Ed Lourens smeared with blood spatters (suspect‐with‐spatters), a wound on his forehead (suspect‐with‐wound), and he is heavily shaking (suspect‐shaking). These findings open up an investigation into what has happened. At this early stage, the space of possibilities is modeled as consisting of one case, expressing the conjunction of the evidence (first row in Fig. 3). In the figure, the evidence encountered is marked on the left, and the empty box indicates the situation to be investigated further. Then the police find the victim’s remains (second row, remains‐victim), implying that the case is now about a killing (victim‐killed). The victim’s face is bloody (victim‐bloody‐face), she lies in a pool of blood (pool‐of‐blood) and there is a visible neck injury (neck‐injury). These cumulative evidential findings (listed on the left side of the figure) suggest that the suspect may be guilty of killing the victim (guilt). But the hypothesis that he is not guilty is also considered a possibility, indicated by the box labeled ­guilt. The two boxes on the third row represent two hypotheses, each a specification of the single hypothesis shown as a box on the top two rows. In other words, the space of possibilities can now be modeled by two cases, expressed by the conjunction of the evidence with either the guilty‐as‐charged hypothesis or the not‐guilty‐as‐charged hypothesis.

In the continued forensic investigation, cartridges are found near the victim’s head and in the vicinity (cartridges), it is established that there is gunshot residue on the victim’s skull (gunshot‐residue‐victim), and there are specific, v‐shaped wounds on the victim’s face (v‐shaped‐wounds). These suggest that the victim was hit by a gun (hit‐by‐gun). It then turns out that the suspect has gunshot residue on his hands and trousers (gun‐shot‐residue‐suspect), that he has threatened the victim multiple times in the past (past‐threats) and that he has been violent towards the victim in the past (past‐violence). These findings indicate that the victim was hit by the suspect (hit‐by‐suspect). It is found that the suspect and victim separated a few months ago (separation) and the victim was dating other men (dating‐other‐men), which provides a motive for the crime (motive).

In the early phases of the investigation, the suspect states (statement‐suspect) that the victim and he were robbed by someone suddenly jumping from the bushes (robbery). This hypothetical scenario is shown on the final row in Fig. 3, with the small box on the right indicating the possibility that there was no robbery as indicated by the suspect and that something else has happened (­robbery, abbreviated in the figure as ­r.). The space of possibilities now consists of three cases, one expressing the guilty‐as‐charged hypothesis, one the robbery scenario, and the third anything else considered possible.

In sum, at the end of these initial findings, a model is constructed consisting of three hypothetical possibilities. The first is the guilty‐as‐charged scenario. In this scenario, the victim is killed (victim‐killed) and the suspect is guilty of killing her (guilt). She was hit by a gun (hit‐by‐gun) by the suspect (hit‐by‐suspect), with the relational problems as motive (motive). The second is the robbery scenario, in which the victim was killed (victim‐killed), the suspect is not guilty (­guilt), and there was a robbery by an unknown man, killing the victim (robbery). The third possibility is that the victim was killed (victim‐killed), the suspect is not guilty ­guilt), and there was also no robbery as described by the suspect (­robbery). Formally, the possibilities are represented as the following three conjunctions:

**Table:** 

1.	victim‐killed ∧ guilt ∧ hit‐by‐gun ∧ hit‐by‐suspect ∧ motive
2.	victim‐killed ∧ ­guilt ∧ robbery
3.	victim‐killed ∧ ­guilt ∧ ­robbery

The collected evidence at this stage is a long conjunction as follows:

**Table:** 

suspect‐with‐spatters ∧ suspect‐with‐wound ∧ suspect‐shaking
∧ remains‐victim
∧ victim‐bloody‐face ∧ pool‐of‐blood ∧ neck‐injury
∧ cartridges ∧ gunshot‐residue‐victim ∧ v‐shaped‐wounds
∧ gunshot‐residue‐suspect ∧ past‐threats ∧ past‐violence
∧ separation ∧ dating‐other‐men
∧ statement‐suspect

Formally, the case model that we have constructed until now consists of three cases, each expressed by one of the hypotheses conjoined with all the evidence. In the model, each of the scenarios is considered to be coherent with the evidence; hence, it is modeled by a case in which the evidence holds. Until now, nothing has been said about their relative plausibility. If we consider the guilty‐as‐charged scenario to be more plausible than the robbery scenario, and that in turn more so than the third, we have a case model in which we have the following:

Case 1 > Case 2 > Case 3

The sizes of the boxes at the bottom row of Fig. 3 have been chosen to fit the corresponding total preorder.

### 3.2 Alternative scenario: A robbery

**Figure 4:**
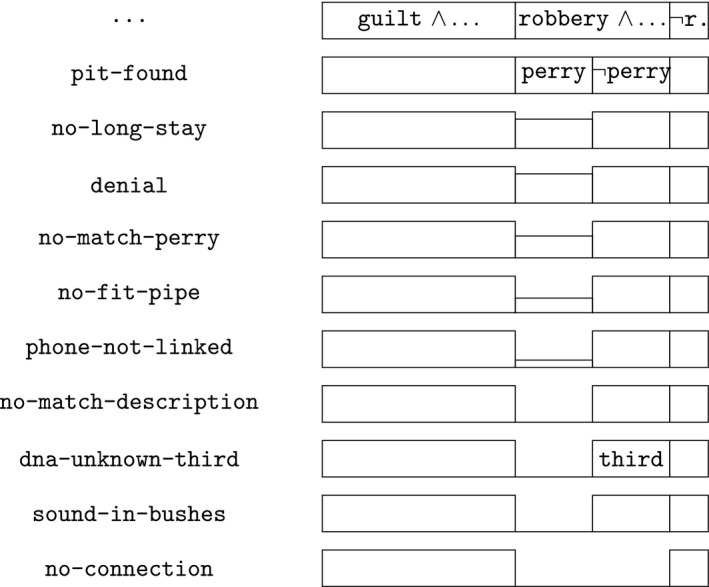
Alternative scenario: A robbery.

After the first court’s decision, the investigation of another, independent case connects Perry Sultan—and perhaps also Tom Seelen, who we ignore in the rest of the text—with the surroundings of the crime scene of the Simonshaven case. In Perry Sultan’s house, the police find a map with marks, one of them close to the place where Jenny was found dead. Subsequently a pit is discovered there (pit‐found). By these findings, the robbery scenario becomes more concrete and is split into two possibilities. In the first possibility, Perry Sultan is the robber jumping from the bushes and killing the victim (perry); in the second, he is not, but someone else is the killing robber (­perry). The findings and options are shown in Fig. 4, second row. The first row corresponds to the end result of the initial findings, as discussed in the previous subsection (Fig. 3).

The possibility that Perry is the killing robber is extensively investigated by the police. First, the pit turns out to be unsuitable for a long stay because of the high groundwater level (no‐long‐stay). Perry denies involvement (denial). The DNA profiles found on the cartridges and clothes and body of the victim do not match Perry’s (no‐match‐perry). The metal pipe used in violent crimes by Perry does not fit the wounds found on Jenny (no‐fit‐pipe). Perry’s phone is not linked to the location (phone‐not‐linked). The description of the robber by Ed as a white man with a bulky stature does not fit Perry, who has southern‐European looks and has a normal to skinny posture (no‐match‐description).

Each of these counts against the scenario that Perry is the robbing killer, except perhaps his denial, which is neutral. Following the outcome of the court proceedings (cf. Section 2.1 and Fig. 2), we model these findings as together excluding the Perry scenario, in the sense that it is no longer a sufficiently reasonable possibility. In Fig. 4, this is indicated by the box for the Perry scenario becoming gradually smaller, until it is reduced to a line on the row labeled no‐match‐description. Since Perry’s denial is neutral evidence for this scenario, the box has equal size on the third and fourth row (labeled no‐long‐stay and denial).

At this stage (modeled by the line no‐match‐perry in Fig. 4), we still have the possibility that someone else than Perry is the killing robber. This possibility becomes more concrete when the DNA of an unknown third person is found on a bullet cartridge (dna‐unknown‐third), suggesting that this third person can be the killer (third). The court considers this finding to not make this possibility more or less believable, as indicated in the figure by a box that has not changed size. Similarly, the rustling sounds in the bushes (sound‐in‐bushes) are modeled as not affecting this possibility. Further investigation does not establish a concrete connection (no‐connection), excluding this possibility. This is shown in Fig. 4 by the second line in the bottom row.

Formally, we start from the case model that we constructed at the end of the previous subsection on the initial findings (Fig. 3), consisting of three cases, one modeling the guilty‐as‐charged scenario, one the robbery scenario, and one the possibility that something else happened. When the pit is found, the case robbery scenario is split into two possibilities. One represents the scenario that Perry Sultan is the killing robber, and the other that an unknown third person is. They are logical specializations of the second case modeling the robbery:

**Table:** 

2.1	victim‐killed ∧ ­guilt ∧ robbery ∧ perry
2.2	victim‐killed ∧ ­guilt ∧ robbery ∧ ­perry

The long conjunction expressing the cumulative evidence considered (established at the end of the previous subsection) is extended with the finding of the pit:

… ∧pit‐found.

The case model corresponding to the second row in Fig. 4 consists of scenarios 1, 2.1, 2.2, and 3, each conjoined with the conjunction of all the evidence. As ordering of the cases, we set:

Case 1 > Case 2.1 ~ Case 2.2 > Case 3

This ordering corresponds to the sizes of the boxes in the figure.

The finding that the found pit is not suitable for a long stay (no‐long‐stay) lowers the plausibility of the scenario about Perry. Formally, the case modeling the scenario is split into two parts, one part in which no‐long‐stay holds, the other ­no‐long‐stay. In the figure, the first part is visible on the third row as a somewhat smaller box than on the second row.

**Table:** 

2.1.1	victim‐killed ∧ ­guilt ∧ robbery ∧ perry
	… ∧ pit‐found
	∧ no‐long‐stay
2.1.2	victim‐killed ∧ ­guilt ∧ robbery ∧ ­perry
	… ∧ pit‐found
	∧ ­no‐long‐stay

Note that Case 2.1.2 is modeled as excluded, but is excluded by the evidence, representing that the believability of the perry‐scenario is reduced. For the ordering, we follow the sizes of the boxes in the figure:

Case 1 > Case 2.2 > Case 2.1.1 > Case 3 > Case 2.1.2

Perry’s denial of being involved extends the cases in the case model with an extra conjunct (… ∧ denial), but does not change the ordering, as it is neutral with respect to the believability of the Perry scenario.

Like the no‐long‐stay evidence, no‐fit‐pipe and phone‐not‐linked lead to a split of the Perry case in a part where the new evidence holds, and where it does not hold. Finally, no‐match‐description excludes the Perry case. For this evidence, there is no split and ­no‐match‐description is taken to hold.

When the DNA of a third unknown person is found (dna‐unknown‐third), the ­perry scenario is specified to include third. This evidence, and also sound‐in‐bushes, does not affect the believability of the Perry scenario. Finally, no‐connection excludes this scenario, formalized by setting that ­no‐connection holds at this stage.

### 3.3 The statement of the suspect

At this stage, two possibilities are still open. The first is the guilty‐as‐charged scenario in which suspect Ed is guilty of killing Jenny. The other is the possibility that something else has happened.

**Figure 5:**
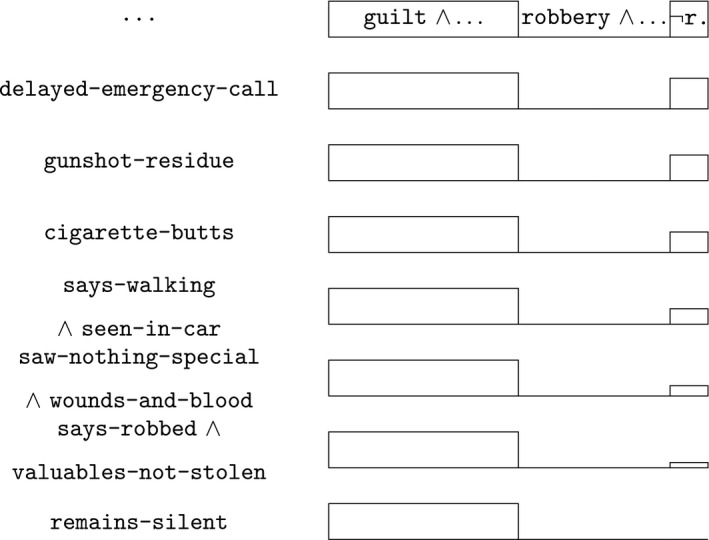
The suspect’s statement.

Following the outcome of the court proceedings, several considerations together exclude the possibility that something else has happened. The development is graphically shown in Fig. 5, modeling the phase in which the suspect’s statement is analyzed.

Several findings remain unexplained by what the suspect has stated—although they could be—which reduces the plausibility of the third alternative that something else has happened. There was a 40‐minute delay before he called his daughter‐in‐law and the emergency services (delayed‐emergency‐call). There is gun‐shot residue on the suspect’s hands and clothes and on the victim’s skull and hands (gunshot‐residue). Three cigarette butts are found next to the victim’s body, and they contain traces with a DNA profile matching the suspect (cigarette‐butts).

Several claims by the suspect are unbelievable or unacceptable considering the other evidence. The suspect says he and the victim were walking (says‐walking), while witnesses saw them in the car (seen‐in‐car). The suspect says he saw nothing special about the victim (saw‐nothing‐special), but she had extensive wounds and there was much blood (wounds‐and‐blood). The suspect says that they were robbed (says‐robbed), while the valuables of suspect and victim were not stolen (valuables‐not‐stolen).

Given all the evidence, the unexplained findings and the unbelievable or unacceptable claims by the victim, he remains silent (remains‐silent).

As shown by its decision, the court takes each of these considerations as making it relatively less believable that something else has happened than that the suspect is guilty, and when all taken together, as making the suspect’s guilt legally and convincingly proven, excluding the possibility that something else has happened. In Fig. 5, this is shown by the boxes on the right gradually becoming smaller, until on the final row at the bottom, it has become a line, indicating that the possibility that something else has happened is excluded.

Formally, the construction is as before in Section 3.22.2.3. The third hypothesis that the suspect is not guilty and there is no robbery, but something else has happened, is at each step split into two, one in which the new evidence holds and one in which it doesn’t. For instance, the rightmost box at the top row corresponds to this hypothesis:

**Table:** 

3.	victim‐killed ∧ ­guilt ∧ ­robbery ∧ …

Here the dots correspond to the conjunction of all the evidential findings taken into account up to this point. If we now consider delayed‐emergency‐call (second row in Fig. 5), the possibility is split into two:

**Table:** 

victim‐killed ∧ ­guilt ∧ ­robbery ∧ … ∧ delayed‐emergency‐call
victim‐killed ∧ ­guilt ∧ ­robbery ∧ … ∧ ­delayed‐emergency‐call

The box in the figure corresponds to the first of these; the second is excluded by the evidence delayed‐emergency‐call.

### 3.4 The suspect’s wounds and his physical capabilities

Two aspects of the case are discussed by the Appellate Court after the analysis of the alternative scenario and statement of the suspect: the suspect’s wounds and the physical capabilities of the suspect. Since at that stage, the robbery scenario and the hypothesis that something else has happened have already been excluded in the model, the logical role of these considerations cannot be properly shown using the case model arrived at at the end of the previous section. Instead, it is more insightful to take a step back, going to the stage where there still are three possibilities: guilt of the suspect, an unexpected robbery, or something else.

**Figure 6:**
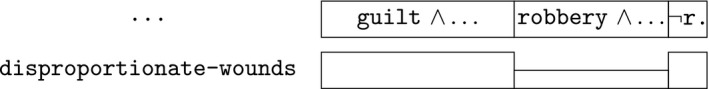
The suspect’s wounds.

First, the court notes that the suspect’s and victim’s wounds are not in proportion (disproportionate‐wounds). The victim has extreme injuries, whereas the suspect has a small wound on his forehead and on the back of his thumb. This fits the guilty suspect scenario, but it goes against the hypothesis of an unexpected robbery, in which the robber attacks both the suspect and the victim. In Fig. 6, this is shown by a change in the size of the box denoting the robbery hypothesis.

**Figure 7:**
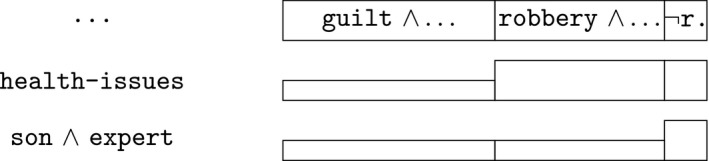
The physical capabilities of the suspect.

Second, the defence has argued that the suspect is not physically capable to apply this kind of force on the victim, given his heart problems and emphysema (health‐issues). This goes against the guilty suspect scenario, as reflected in Fig. 7 by the smaller box on the second row. This point by the defence is countered by a statement by the suspect’s son that the suspect’s capabilities are good when he has taken his medication (son). Also, an expert reports that, despite his health issues, the suspect was physically sufficiently capable to apply the kind of force assumed in the guilty suspect scenario. In Fig. 7, this is reflected by reducing the size of the box for the robbery hypothesis relative to that for the guilty‐as‐charged scenario to the same level as before considering the health issues. In the figure, they are shown with equal size boxes on the third row, just as on the first row.

## 4 The full case model, with connections to arguments, scenarios, and probabilities

**Figure 8:**
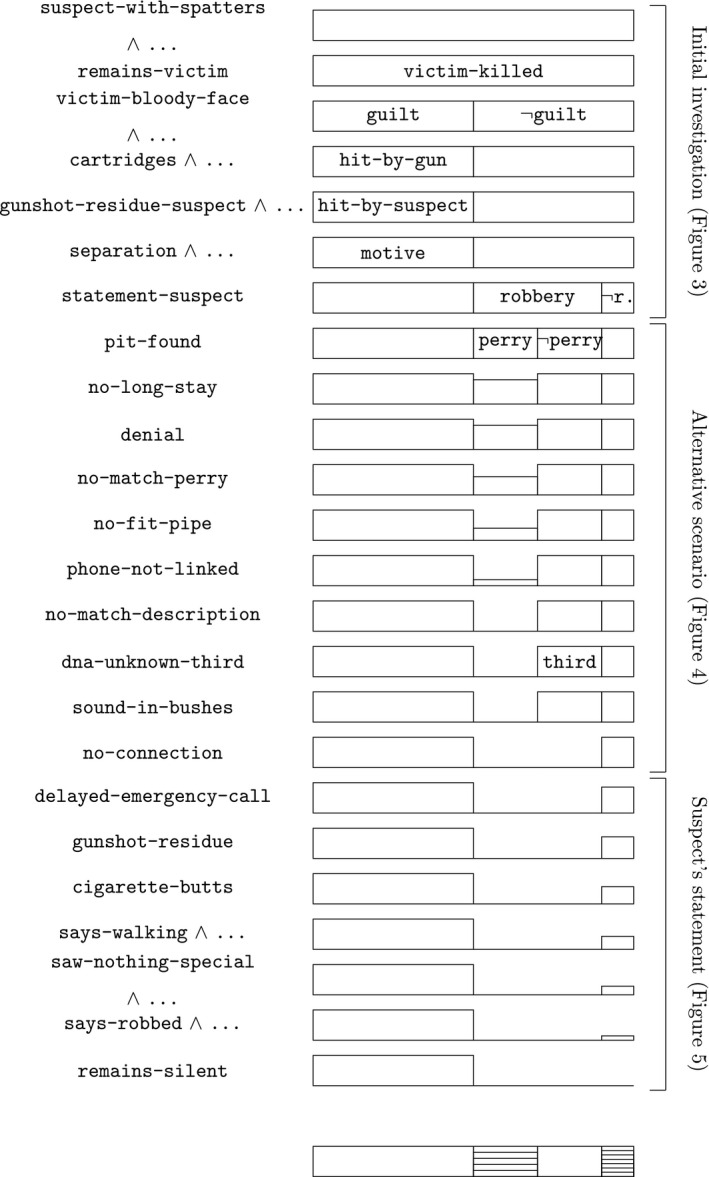
Analysis of the Appellate Court’s reasoning; with the structure of the resulting case model at the bottom.

Based on its analysis of the evidence and the hypotheses, the Appellate Court decides that the crime is proven because of the facts and circumstances that follow from the evidence and because of the lack of a reasonable explanation for several circumstances. The court also considers that it has not become reasonable that someone else committed the crime; hence, it concludes that it has been proven lawfully and convincingly that the suspect committed manslaughter. The full analysis is shown in Fig. 8.

The resulting full case model’s structure is shown at the bottom of Fig. 8, and graphically it is arrived at by positioning all rows in the analysis above over one another.

The four core hypotheses (specifications of the four options discussed in Section 22) are as follows. The phrases in capitals are used as abbreviating place‐holders below.

**Table:** 

1.	GUILT: victim‐killed ∧ guilt ∧ hit‐by‐gun ∧ hit‐by‐suspect ∧ motive
2.	PERRY: victim‐killed ∧ ­guilt ∧ robbery ∧ perry
3.	THIRD: victim‐killed ∧ ­guilt ∧ robbery ∧ ­perry ∧ third
4.	OTHER: victim‐killed ∧ ­guilt ∧ ­robbery

The totality of the evidence is collected in a long conjunction, as follows.

**Table:** 

EVIDENCE:
suspect‐with‐spatters ∧ suspect‐with‐wound ∧ suspect‐shaking
∧ remains‐victim
∧ victim‐bloody‐face ∧ pool‐of‐blood ∧ neck‐injury
∧ cartridges ∧ gunshot‐residue‐victim ∧ v‐shaped‐wounds
∧ gunshot‐residue‐suspect ∧ past‐threats ∧ past‐violence
∧ separation ∧ dating‐other‐men
∧ statement‐suspect
∧ pit‐found ∧ no‐long‐stay ∧ denial ∧ no‐match‐perry ∧ no‐fit‐pipe
∧ phone‐not‐linked ∧ no‐match‐description
∧ dna‐unknown‐third ∧ sound‐in‐bushes ∧ no‐connection
∧ delayed‐emergency‐call ∧ gunshot‐residue ∧ cigarette‐butts
∧ says‐walking ∧ seen‐in‐car
∧ saw‐nothing‐special ∧ wounds‐and‐blood
∧ says‐robbed ∧ valuables‐not‐stolen
∧ remains‐silent

In the following, initial parts of the EVIDENCE conjunction are denoted EVIDENCE(<sentence>), where the conjunction ends at <sentence>. For instance, EVIDENCE(remains‐victim) equals suspect‐with‐spatters ∧ suspect‐with‐wound ∧ suspect‐shaking ∧ remains‐victim.

Then the cases of the case models are as follows.

**Table:** 

Case 1: GUILT ∧ EVIDENCE
Case 2.1: PERRY ∧ EVIDENCE(pit‐found) ∧ ­no‐long‐stay
Case 2.2: PERRY ∧ EVIDENCE(denial) ∧ ­no‐match‐perry
Case 2.3: PERRY ∧ EVIDENCE(no‐match‐perry) ∧ ­no‐fit‐pipe
Case 2.4: PERRY ∧ EVIDENCE(no‐fit‐pipe) ∧ ­phone‐not‐linked
Case 2.5: PERRY ∧ EVIDENCE(phone‐not‐linked) ∧ ­no‐match‐description
Case 3: THIRD ∧ EVIDENCE(sound‐in‐bushes) ∧ ­no‐connection
Case 4.1: OTHER ∧ EVIDENCE(no‐connection) ∧ ­delayed‐emergency‐call
Case 4.2: OTHER ∧ EVIDENCE(delayed‐emergency‐call) ∧ ­gunshot‐residue
Case 4.3: OTHER ∧ EVIDENCE(gunshot‐residue) ∧ ­cigarette‐butts
Case 4.4: OTHER ∧ EVIDENCE(cigarette‐butts) ∧ ­(says‐walking ∧ seen‐in‐car)
Case 4.5: OTHER ∧ EVIDENCE(says‐walking ∧ seen‐in‐car) ∧ ­(saw‐nothing‐special ∧ wounds‐and‐blood)
Case 4.6: OTHER ∧ EVIDENCE(saw‐nothing‐special ∧ wounds‐and‐blood) ∧ ­(says‐robbed ∧ valuables‐not‐stolen)
Case 4.7: OTHER ∧ EVIDENCE(says‐robbed ∧ valuables‐not‐stolen) ∧ ­remains‐silent

The ordering relation on these fourteen cases is as follows. The ordering is chosen as fitting the sizes of the boxes in the case model at the bottom of Fig. 8.

Case 1 > Case 3 > Case 2.1 ∼ ⋯ ∼ Case 2.5 > Case 4.1 ∼ ⋯ ∼ Case 4.7

As in Section 2.22.2, the case model can be connected to arguments, scenarios, and probabilities.

### 4.1 Arguments

Arguments from premises to conclusions can be evaluated in the case model. For instance, the argument from premise remains‐victim to conclusion victim‐killed is coherent (by Case 1), presumptively valid (since Case 1 is maximal in the ordering), and conclusive (since all cases imply victim‐killed). The argument from pit‐found to perry is coherent (for instance by Case 2.1) and not presumptively valid (since Case 1 implies pit‐found but is higher in the ordering than cases that imply perry, such as Case 2.1). The argument is also not conclusive since there are cases implying the premise that do not imply the conclusion (such as Case 1 and Case 3).

The case model shows defeating circumstances for arguments. For instance, the coherent argument from pit‐found to perry is no longer coherent when the premises no‐long‐stay, …, no‐match‐description are added since there is no case that implies both the extended argument’s premises pit‐found ∧ no‐long‐stay ∧ … ∧ no‐match‐description and its conclusion perry.

The case model focuses on the court’s reasoning and does not represent all argumentative aspects of the case. For instance, in the case model, the argument from motive to guilt—which seems reasonable—has no special role. It is coherent, but that is a very weak measure of validity.

### 4.2 Scenarios

In the analysis of the case, gradually ever more specific scenarios about what has happened are constructed. In the full case model, in particular the guilty‐as‐charged scenario has been further specified: The victim was killed (victim‐killed), hit by a gun (hit‐by‐gun), and hit by the suspect (hit‐by‐suspect), who had a motive for killing the victim (motive). The other scenarios remain less specific in the analysis. In the Perry robbery scenario, the victim is killed (victim‐killed), in a robbery (robbery), by Perry (perry).

The least specific scenario hardly deserves that name: It is the “scenario” that something else than what is considered has happened. In that scenario, the victim is killed (victim‐killed), but not according to the guilty‐as‐charged scenario (­guilt) and not in a robbery (­robbery).

In the case model, the guilty‐as‐charged scenario follows conclusively from the evidence and hence is beyond a reasonable doubt given all the evidence. The other three considered scenarios are coherent, but they are not coherent given all the evidence.

The case model has a focus on the global coherence of scenarios, in the sense that cases model whether the conjunction of the events in a scenario is considered possible. The local coherence of scenarios, for instance, internal causal connections (motive is a causal factor leading to victim‐killed) or the time ordering (hit‐by‐gun precedes victim‐killed) is not considered.

### 4.3 Probabilities

As in Section 2.22.2, the case model’s ordering can be realized probabilistically. For instance, we can as before use the relative sizes of the boxes in the diagram at the bottom of Fig. 8 (though many other numeric realizations are possible). This leads to the following (approximate) probabilities for the 14 cases: Case 1, Case 3, Case 2.1, …, Case 2.5, Case 4.1, …, Case 4.7, respectively: 50%, 20%, 4%, …, 4%, 1.4%, …, 1.4%. Coherent arguments correspond to positive (conditional) probability (representing argument strength) and conclusive probability to 100% strength. Possibilities are excluded when they have a probability of 0%. For instance, before the evidence the guilty‐as‐charged scenario has 50/(50 + 20 + 4 + ⋯ + 4 + 1.4 + ⋯ + 1.4) = 50% probability. The argument from empty premises to perry has strength (4 + ⋯ + 4)/100 = 20%. Given all the evidence, the guilty‐as‐charged scenario has 100% probability, and the other possibilities 0%.

The case model focuses on the ordering of probabilities, and on the extreme values of 0% and 100%. Specific numeric effects are not modeled. For instance, here the Perry‐robbery scenario is modeled as being equivalent in the ordering to the third‐person‐robbery scenario. In the numeric interpretation, when the Perry robbery scenario is excluded, the (relative) probability of the third‐person‐robbery scenario increases. Using the numbers above, we have that guilt: perry: third: “something else” changes from 50: 20: 20: 10 to 50: 0: 20: 10, so the third‐person‐robbery scenario grows from 20/100 = 20% probable to 20/80 = 25% probable. The something‐else scenario changes from 10% to 12.5%. If one assumes that the third‐person scenario should not or negligibly be affected by the exclusion of the Perry scenario, different modeling choices should be made.

## 5 Discussion of the analysis using the four guiding questions

We discuss the analysis of the Simonshaven case using four guiding questions that were suggested by the special issue editors.

- **Objectivity and subjectivity**. To what extent is the analysis objective and to what extent is it based on subjective beliefs, assumptions, and choices?

The outcome of the analysis can be considered as a model of the Appellate Court’s reasoning, as they appear in the source material available. The intention has been to stay as close as possible to the sources, without adding new elements. Choices have been made in the selection of which elements of the discussions are modeled, and how they are structured. The case model approach does not require the specification of specific probabilities, hence limiting the assumptions that to be made in addition to what is available in the source material. Assumptions are made concerning the ordering of the cases in the case model. Their specific sizes in the graphical representations may suggest more specific assumptions than are modeled in the orderings, but they do not affect the analysis of how the Appellate Court arrives at its final decision. For evaluative parts of the analysis (including the choice of the ordering), we have focused on the summaries and decisions in the Appellate Court’s reasoning, in particular also for the final decision about whether guilt has been proven legally and convincingly. In this way, we address the issue that in hard cases it can be subject to debate “what counts as enough” for establishing guilt, and leave it to the court that has the authority to decide the debate. As the model aims to follow the court’s decision, the debate can then continue about whether that model is as it should be.

- **Cognitive and legal naturalness**. How natural is the analysis from a cognitive and legal point of view?

The analysis of the Appellate Court’s reasoning (as summarized in Fig. 8) is natural in the sense that it can be read as a model of the investigation of the case, in which gradually hypotheses are developed and evaluated, guided by a sequence of evidence taken into account. Arguments and scenarios associated with the analysis are close to their counterparts in human reasoning and legal practice. Whereas probabilities are sometimes considered to be less natural from a human reasoning and legal practice perspective, in particular since people often make errors and it can be hard to assign all relevant numbers, here probabilities play a role in the background. Still the method adheres to the probability calculus, in the sense that case models have a probabilistic interpretation, hence preventing probabilistic reasoning errors. Errors can of course still be made, but then the errors are part of the model, and can be corrected by adapting the model. Also as said there was no need in our analysis to assign specific numbers, instead focusing on whether a probability is positive or equal to one, how the probabilities are ordered, and how these develop under conditionalization by additional evidence. In this way, there is more emphasis on the qualitative aspects of probabilities while staying within the bounds of Bayesian rationality.

By this mix of argumentative, scenario, and probabilistic methods of analysis, an attempt has been made to balance the strengths of each.

- **Errors and biases**. Did your analysis identify errors or biases in the reasoning of the judge, prosecutor, or defence?

The analysis aims to identify the assumptions that are needed for arriving at a decision. These assumptions are encoded in the case model that is the result of the analysis. The focus has been on the reasoning of the Appellate Court. The analysis suggests that the court has arrived at a balanced decision, using the material brought forward by prosecutor or defence. In one detail, namely the role of Perry’s denial that he is the robber, our analysis models this evidence as neutral with respect to the robbery scenario, where the court seems to consider it as evidence, making the robbery scenario more strongly supported. This choice plays no role in the overall outcome of the decision.

- **Legal constraints**. Does your analysis respect the legal constraints, such as the burden and standard of proof and the right to remain silent?

Our approach aims to provide a rational analysis of the evidence, the hypotheses, and their evaluation. Legal constraints are not an explicit part of the approach. Still specific themes connected to legal constraints can be addressed. We discuss specific legally relevant themes: the presumption of innocence, the standard of proof, the burden of proof, and the right to remain silent. The presumption of innocence is addressed in our approach as initially the not‐guilty‐as‐charged scenario is considered a coherent possibility, to be excluded on the basis of specific evidence. As standard of proof, we consider a position to be legally and convincingly proven when it follows conclusively (using qualitative terminology) or, equivalently, when it follows with probability 1 (using quantitative terminology). As explained in Verheij (2014), this allows for a distinction between reasonable and unreasonable doubt: Doubt about a position is reasonable when there is an alternative position that is considered possible; doubt is unreasonable when there is no such position. All remaining doubt—inevitably always there—is transferred to doubt about the model, for instance, about whether all possibilities have been taken into account. This is a modeling choice. It is also possible to always model an alternative hypothesis of minimal plausibility (minimal in the ordering) that represents remaining doubt. With respect to the burden of proof, our method of analysis allows for the inclusion of all discussion elements brought forward. By including the not‐guilty‐as‐charged possibility from the start, the prosecution has the burden to exclude that possibility by additional evidence, while the defence does not have to provide a complete alternative scenario. With respect to the right to remain silent, our approach is neutral. What is not said is not in the model. In this case, the Appellate Court’s reasoning included the silence of the suspect as counting against him. In our analysis, the suspect’s silence has therefore been explicitly modeled (remains‐silent).

## 6 Conclusion

In sum, we have provided an analysis of the Simonshaven case, using a method “with and without probabilities”: It is with probabilities, in the sense that the analysis adheres to the probability calculus and can be given a numeric probabilistic interpretation. It is without probabilities, in the sense that the analysis does not require the specification of many numbers. Characteristics of the approach are as follows:

1. *A combination of theory construction and evidential update*. The end result of the analysis is a case model that is gradually constructed, in a process similar to an investigation. Hypotheses are developed and evaluated under the influence of a sequence of evidence.
2. *A combination of qualitative and quantitative modeling*. Case models consist of formal cases and a preference ordering; hence, they are qualitative in nature, but can be given a quantitative interpretation, adhering to the probability calculus.
3. *A combination of arguments, scenarios, and probabilities*. Arguments and scenarios can be evaluated in the case model. Arguments can be coherent, presumptively valid and conclusive. Arguments can have defeating circumstances. Scenarios can be coherent, plausible, and beyond a reasonable doubt. These notions can be given a probabilistic interpretation.

Compared to existing argumentative, scenario, and probabilistic approaches, the approach has several limitations. Argumentative approaches extensively study argument structure and its connection to argument evaluation, where the present approach is based on notions of formal validity of elementary arguments and defeating circumstances. In scenario approaches, the role of causal and evidential generalizations is studied as tools for assessing scenario anchoring and quality, a topic remaining unaddressed in the present approach. Probabilistic approaches have a precise numerical theory of the interaction between several pieces of evidence and hypothetical events, while the present approach focuses on the ordering of cases only. Also, in argumentative, scenario, and probabilistic approaches, significant attention is paid to knowledge representation aspects, concretely by the study of argumentation schemes, scenario schemes, and BN idioms. Such tools for knowledge representation have here not been addressed.

Following the initial proposal of the case model approach “with and without probabilities” and its applications to evidential reasoning (Verheij, 2014, 2017), this study is the first in which it has been applied to a real‐world case in full complexity.

In some respects, the results seem promising. For instance, the full analysis of the case (as graphically represented in Fig. 8) models significant parts of the court’s reasoning, while staying reasonably close to what is actually said in the decision text. No specific numeric probabilistic estimates are needed for showing how the reasoning develops, while considerations of relative plausibility are modeled. Alternative hypotheses, among them the scenarios considered by the court, are explicitly available and modeled in their connection to the evidence. The case model construction shows which evidence supports or excludes which hypotheses. In these ways, elements of probabilistic, scenario, and argumentative analyses appear side by side (see Di Bello & Verheij, 2018 for an exposition of the related, yet different roles played by probabilities, scenarios and arguments in evidential reasoning). Hence the two issues discussed about Bayesian network modeling (Section 11) are addressed: the issue of incompleteness of numeric information since case models require no specific numeric estimates; and the issue of transparent interpretation since case models are formally connected to probabilistic, argumentative, and scenario notions.

In other respects, questions remain. For instance, could a fine‐grained probabilistic, argumentative, or scenario model be translated into a case model? Which elements can be kept, and which are lost? Where does the ordering of the cases come from? Are there systematic ways of developing such an ordering, perhaps based on the court’s decision text directly, or by using translations from other kinds of analysis? Aren’t there hidden assumptions that hinder modeling or imply reasoning errors?

Further research may help answer such questions, and comparing the results of the different analyses in this special issue would be a valuable tool for making progress. Thereby, we would gradually develop our understanding of how probabilistic, argumentative, and scenario analytic methods are connected and can strengthen one another.
